# Role of blood Krebs von Lungen-6 in predicting acute exacerbation in patients with idiopathic pulmonary fibrosis

**DOI:** 10.1371/journal.pone.0323784

**Published:** 2025-05-30

**Authors:** Eun Jun Choe, Ji Hoon Jang, Jin Han Park, So Young Jung, Minyoung Her, Jae Ha Lee

**Affiliations:** 1 Division of Pulmonary and Critical Care Medicine, Department of Internal Medicine, Inje University Haeundae Paik Hospital, Inje University College of Medicine, Busan, Republic of Korea; 2 Department of Dermatology, Inje University Haeundae Paik Hospital, Inje University College of Medicine, Busan, Republic of Korea; 3 Division of Rheumatology, Department of Internal Medicine, Inje University Haeundae Paik Hospital, Inje University College of Medicine, Busan, Republic of Korea; Kurume University School of Medicine: Kurume Daigaku Igakubu Daigakuin Igaku Kenkyuka, JAPAN

## Abstract

**Background:**

This study evaluated the role of blood Krebs von den Lungen-6 (KL-6) in predicting acute exacerbation (AE) in patients with idiopathic pulmonary fibrosis (IPF).

**Methods:**

From April 2018 to March 2023, clinical data of 233 IPF patients with baseline and follow-up KL-6 values at Haeundae Paik Hospital were retrospectively analyzed. AE was defined following the criteria proposed by Collard et al. in 2016.

**Results:**

The mean age was 71.8 years; 79% were male. During follow-up (median: 18.7 months), 33 (14.2%) patients experienced AE. Throughout the entire period from baseline, KL-6 values were higher in the AE group compared to the non-AE group (*P* < 0.001), and the patterns of change over time also showed significant differences between both groups (*P* < 0.001). The KL-6 values in the post-exacerbation phase were higher than those in the pre-exacerbation phase among the AE group (*P* = 0.004). The AE group showed lower 1-year (86.4% vs. 95.9%) and 3-year (50.2% vs. 91.4%) survival rates compared to the non-AE group (*P* < 0.001). The occurrence of AE (hazard ratio (HR) 74.09, 95% confidence interval (CI) 31.97–171.7, P < 0.001) and higher lactate dehydrogenase (HR 1.02, 95% CI: 1.01–1.02, *P* < 0.001) were independently associated with mortality in patients with IPF

**Conclusions:**

Our data suggest that the trend in changes in KL-6 values may be utilized as a tool for predicting AE-IPF. Further research is needed to establish the clinical significance of changes in KL-6 for predicting AE-IPF and to validate the cut-off values for prediction.

## Introduction

Idiopathic pulmonary fibrosis (IPF) is a form of chronic and progressive fibrosing interstitial lung disease (ILD), characterized by a poor prognosis with a median survival time of 2–5 years [1–3]. In the heterogeneous disease course, acute exacerbation (AE) of the IPF (AE-IPF) is a critical event and a major cause of death in affected patients [4]. The annual incidence of AE in patients with IPF has been reported as 5–15%, depending on the patient population and the definition of AE [5,6]. The mortality rate of AE-IPF is known to be approximately 50%, and in severe cases that need mechanical ventilators, the in-hospital mortality rate has been reported to reach 87% [7–9]. Despite the importance of AE-IPF and medical advancements in the field of IPF over the past decades, there are no proven treatments other than corticosteroids [10]. Therefore, it is crucial to identify the risk factors associated with AE for early prediction and appropriate diagnosis of AE-IPF.

Some risk factors for AE-IPF have been reported[11–13]. Advanced disease state with low forced vital capacity (FVC) and diffusion capacity of the lungs for carbon monoxide (DLco), shorter six-minute walking distance (6MWD), and impaired oxygenation were the most important risk factors for AE-IPF in previous studies [14–18]. Previous history of AE and a recent decline in FVC were also reported as risk factors [19,20]. Recently, blood biomarkers, including Krebs von den Lunger-6 (KL-6), have gained attention for their objectivity, ease of evaluation, and good reproducibility for predicting AE and prognosis [21]. KL-6 is a mucin-like, high-molecular-weight glycoprotein produced in the lungs by injured and regenerating type 2 epithelial cells and has been widely used in clinical practice in patients with IPF [22,23]. A previous study reported that baseline KL-6 values were useful for predicting AE-IPF in 77 patients with IPF [24,25]. Although some studies suggest that serial changes in KL-6 may predict disease progression in ILD [26,27], there is a lack of evidence examining the role of changes in KL-6 in predicting AE-IPF. Therefore, this study aimed to analyze the clinical role of changes in KL-6 for predicting the occurrence of AE-IPF and prognosis.

## Materials and methods

### Study design and population

**Fig 1:**
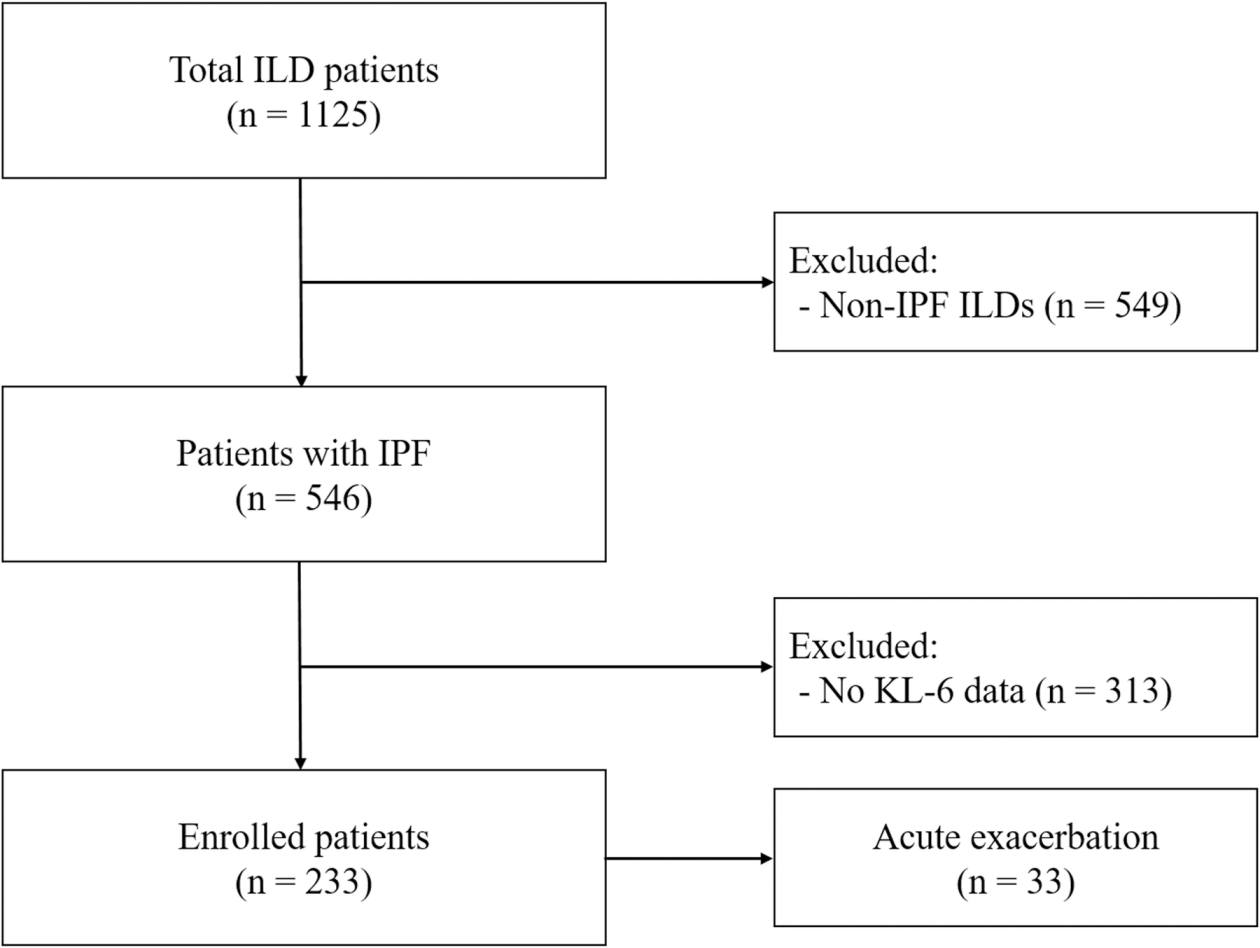
Flow chart of patient selection. ILD, interstitial lung disease; IPF, idiopathic pulmonary fibrosis; KL-6, Krebs von den Lungen-6.

From April 2018 to March 2023, 1125 patients who were diagnosed with ILD at Haeundae Paik Hospital in Republic of Korea, were screened for this study. The investigation of the medical records was conducted for three weeks, from April 3 to April 29, 2023. Of the 1125 patients with ILD, 549 were excluded due to non-IPF diagnosis. Finally, 233 IPF patients with available data of KL-6 blood tests conducted at baseline and follow-up visits were included in this study (Fig 1). IPF was diagnosed using the criteria of the 2022 American Thoracic Society (ATS)/European Respiratory Society (ERS)/Japanese Respiratory Society (JRS)/Latin American Thoracic Association (ALAT) statement [10]. AE was defined following the diagnostic criteria suggested by Collard et al., as acute respiratory deterioration typically < 1-month duration, with new bilateral lung infiltration on computed tomography that is not fully explained by cardiac failure or fluid overload without alternative diagnosis (pneumothorax, pleural effusion, or pulmonary embolism) [28].

This study was approved by the Institutional Review Board of Haeundae Paik Hospital (approval no. 2022-12-023) and was conducted in accordance with the ethical standards of the Declaration of Helsinki. The requirement for written informed consent to participate was waived due to the retrospective nature of this study. Furthermore, obtaining consent for publication was also waived due to the retrospective nature of the study.

### Data collection

The clinical and survival data for all enrolled patients were obtained retrospectively from medical records. Since the investigation involved actual patients’ medical records with identifiable information, personal identifiers such as medical record numbers and names were removed and anonymized in the preparation of the data set. The baseline value of KL-6 was the first measured value of KL-6 without evidence of AE after diagnosis of IPF. Spirometry DLco were measured according to the ERS/ATS recommendations every six months; the results are presented as percentages of the normal predicted values with the corrected DLco for hemoglobin [29–31]. The results of the six-minute walk test (6MWT) were expressed according to the previously published guidelines [32].

Laboratory tests including KL-6 were measured at baseline and every three months at follow-up visits. Furthermore, in the AE group, measurements of KL-6 were conducted at weekly intervals following the diagnosis of AE-IPF continuing until either clinical improvement or the occurrence of mortality. The Nanopia KL-6 assay (SEKISUI MEDICAL, Tokyo, Japan) was used for analysis of blood KL-6 values.

### Outcomes

The primary outcome was to analyze the changes in KL-6 values between the AE group and the non-AE group to determine the clinical significance of changes in KL-6 for prediction of AE. The secondary outcomes included: 1) a comparison of KL-6 values before and after AE, 2) a survival analysis of the AE group and the non-AE group, and 3) the identification of predictors for mortality in patients with IPF.

### Statistical analysis

Variables are presented as frequency and percentage for categorical data and mean ± standard deviation and median (IQR) for numeric data. Group differences were tested using the chi-square test or Fisher’s exact for categorical data and independent t test or Mann–Whitney U test for numeric data as appropriate. To check normal distribution of data, we used the Shapiro–Wilk’s test. To assess the trends in KL-6 values between the acute exacerbation (AE) and non-AE groups, a generalized linear mixed model (GLMM) with random intercepts was employed. For the AE group, KL-6 values recorded at three-month intervals leading up to the onset of the AE were analyzed, while for the non-AE group, all KL-6 values measured at three-month intervals were included in the analysis. In the AE group, the absolute difference between baseline KL-6 values and those measured immediately before the acute exacerbation was incorporated as a variable, and subsequent binary logistic regression was utilized for both univariate and multivariate analyses to identify independent risk factors associated with the occurrence of the AE. In addition, the changes in KL-6 values before and after the AE were analyzed using Wilcoxon’s signed-rank test. Overall survival probability was estimated by the Kaplan–Meier method. The difference between survival rates was assessed using the log-rank test. The disease interval was measured from the day of diagnosis until death or the most recent follow-up. Death from all causes was included. Univariate and multivariate Cox proportional hazards models were fit to examine the relationship between patient survival and characteristics.

All statistical analyses were performed using SPSS 26.0 (IBM Corp. Released 2019. IBM SPSS Statistics for Windows, Version 26.0. Armonk, NY: IBM Corp), R 4.1.2 (R Core Team (2021). R: A language and environment for statistical computing. R Foundation for Statistical Computing, Vienna, Austria. URL https://www.R-project.org/), and MedCalc Statistical Software version 19.2.6 (MedCalc Software Ltd, Ostend, Belgium; https://www.medcalc.org; 2020). A p-value less than 0.05 was considered statistically significant.

## Results

### Study population

Of the 233 enrolled patients, 79.0% were male (Table 1). The mean patient age was 71.8 years, and 77.6% were ever smokers. Most patients showed mild restrictive lung function defect of FVC (75.0, % predicted) and (82.8%) received treatment with antifibrotic agents with a median treatment duration of 14.6 months. During follow-up (median: 18.7 months, interquartile range 6.1–38.7 months), 33 (14.2%) patients experienced AEs. The AE group showed lower body mass index (BMI), lower DLco, lower saturation of peripheral oxygen (SpO_2_) during 6MWT, higher KL-6 at baseline, and higher proportion of home oxygen use.

**Table 1:** Comparison of characteristics between IPF patients with and without AE.

	Overall	AE	non-AE	*P*-value
Number of patients	233 (100.0)	33 (14.2)	200 (85.8)	
Age, years	71.8 ± 8.1	72.0 ± 8.7	71.7 ± 8.1	.870
Male	184 (79.0)	26 (78.8)	158 (79.0)	.978
Ever smokers	177 (77.6)	25 (75.8)	152 (77.9)	.780
BMI, kg/m^2^	24.6 ± 3.1	23.4 ± 3.1	24.8 ± 3.1	.034
PFT, FVC, % predicted	75.0 ± 16.6	71.9 ± 21.3	75.5 ± 15.6	.350
PFT, DLco, % predicted	59.2 ± 16.6	47.8 ± 16.9	61.1 ± 15.9	<.001
6MWD, m	434.5 ± 105.3	404.0 ± 119.3	439.1 ± 102.6	.100
6MWT, Baseline SpO_2_, %	96.0 ± 2.6	94.0 ± 3.8	96.3 ± 2.3	<.001
6MWT, Lowest SpO_2_, %	90.0 ± 7.0	84.0 ± 9.9	90.9 ± 6.0	<.001
WBC, 10^3^/μL	7.8 ± 2.5	8.9 ± 2.6	7.6 ± 2.4	.010
LDH, IU/L	238.5 ± 62.3	245.9 ± 55.3	237.2 ± 63.5	.217
CRP, mg/dL	1.2 ± 3.0	0.6 ± 0.7	1.3 ± 3.3	.879
BNP, pg/mL	864.9 ± 3492.5	1179.0 ± 3796.5	793.1 ± 3429.7	.836
Baseline KL-6, U/mL	855.7 ± 658.4	1188.9 ± 767.0	800.7 ± 623.9	<.001
GAP stage				.013
Stage I	116 (49.8)	9 (27.3)	107 (53.5)	
Stage II	95 (40.8)	19 (57.6)	76 (38.0)	
Stage III	22 (9.4)	5 (15.2)	17 (8.5)	
Home oxygen use	48 (20.8)	26 (81.3)	22 (11.1)	<.001
Antifibrotics use	193 (82.8)	32 (97.0)	161 (80.5)	.020

Values are presented as mean ± standard deviation or number (%).

IPF, idiopathic pulmonary fibrosis; AE, acute exacerbation; BMI, body mass index; PFT, pulmonary function test; FVC, forced vital capacity; DLco, diffusing capacity of the lungs for carbon monoxide; 6MWD, six-minute walking distance; 6MWT, six-minute walk test; SpO_2_, saturation of peripheral oxygen; WBC, white blood cell; LDH, lactate dehydrogenase; CRP, C-reactive protein; BNP, brain natriuretic peptide; KL-6, Krebs von den Lungen-6; GAP, gender, age, and physiology

### Trends in KL-6 values over time

**Fig 2:**
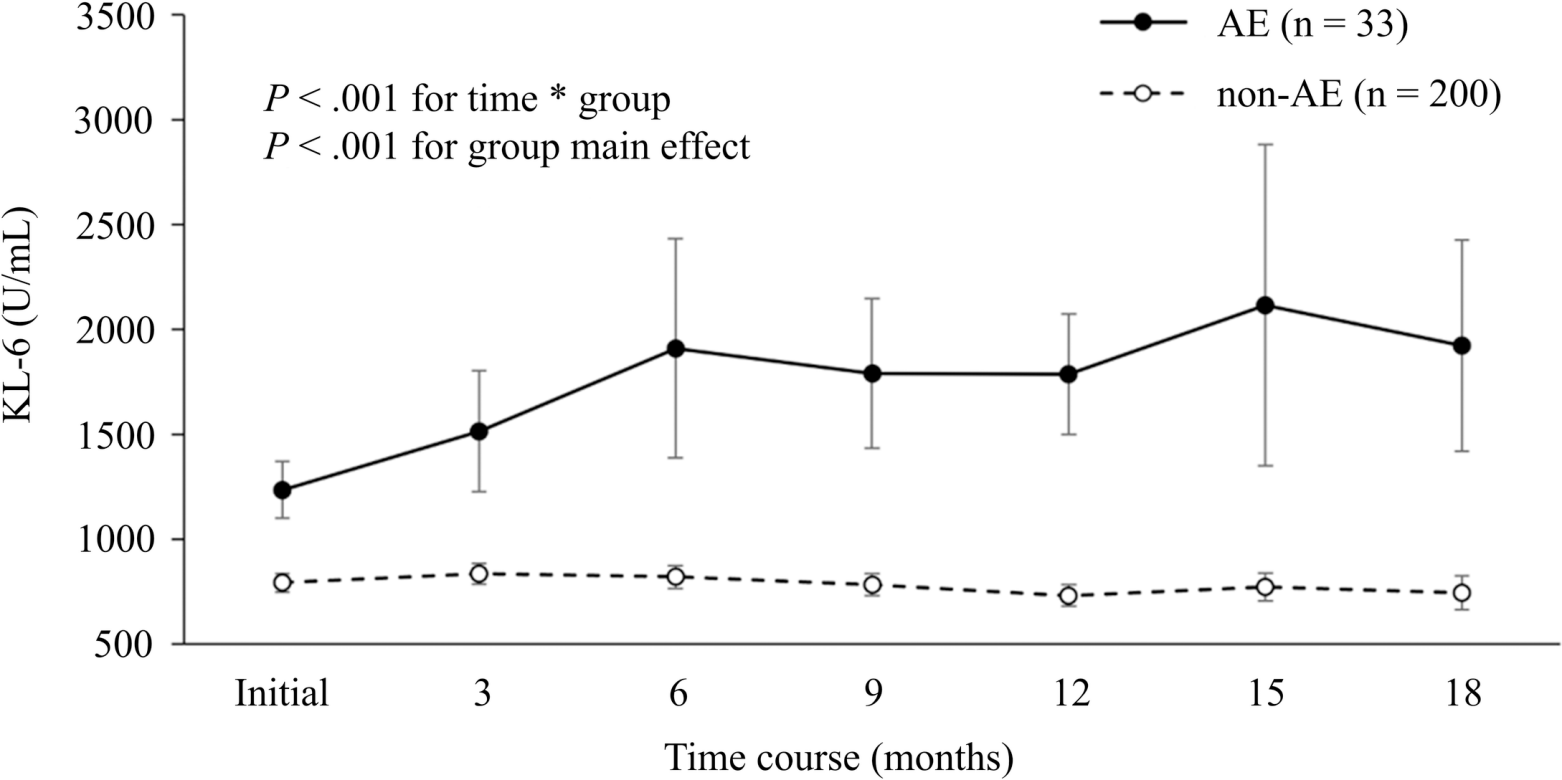
Comparison of the trends in KL-6 values over time between the non-AE group and the pre-exacerbation phase of the AE group. P-value for ‘time*group’ represents interaction between group and time. KL-6, Krebs von den lungen-6; AE, acute exacerbation.

Analysis of KL-6 values over time in both the AE and non-AE groups indicated that the AE group exhibited significantly higher mean KL-6 values than the non-AE group across all intervals from baseline to 18 months (Table 2). A comparative analysis of the trends in KL-6 changes over time between the two groups, conducted using a GLMM, demonstrated a significant group main effect (*P* < 0.001). Additionally, the interaction between group and time, which highlights significant differences in the patterns of change in KL-6 over time, also reached statistical significance (*P* < 0.001). KL-6 values showed an upward trend during the pre-exacerbation phase in the AE group, while the non-AE group exhibited no distinct trends in changes (Fig 2).

**Table 2:** Time-course changes in KL-6 values between the AE group and the non-AE group.

Time	Group			Analysis for repeated measures	
AE (n = 33)	non-AE (n = 200)	*P*-value	Source	*P*-value
Initial	1,235.4 ± 772.3	793.1 ± 618.7	<.001	Group	<.001
at 3 months	1,515.5 ± 1,258.6	836.2 ± 615.3	<.001	Time	<.001
at 6 months	1,910.9 ± 1,386.1	821.2 ± 623.8	.002	Time*Group	<.001
at 9 months	1,790.5 ± 939.4	783.3 ± 562.0	<.001		
at 12 months	1,786.5 ± 495.0	733.0 ± 486.2	.007		
at 15 months	2,116.9 ± 1,325.5	773.0 ± 592.7	.014		
at 18 months	1,924.5 ± 712.7	745.9 ± 612.3	.036		

P-value for Time*Group represents interaction between group and time

KL-6, Krebs von den lungen-6; AE, acute exacerbation

### Changes in KL-6 values in the pre- and post-AE phases

**Fig 3:**
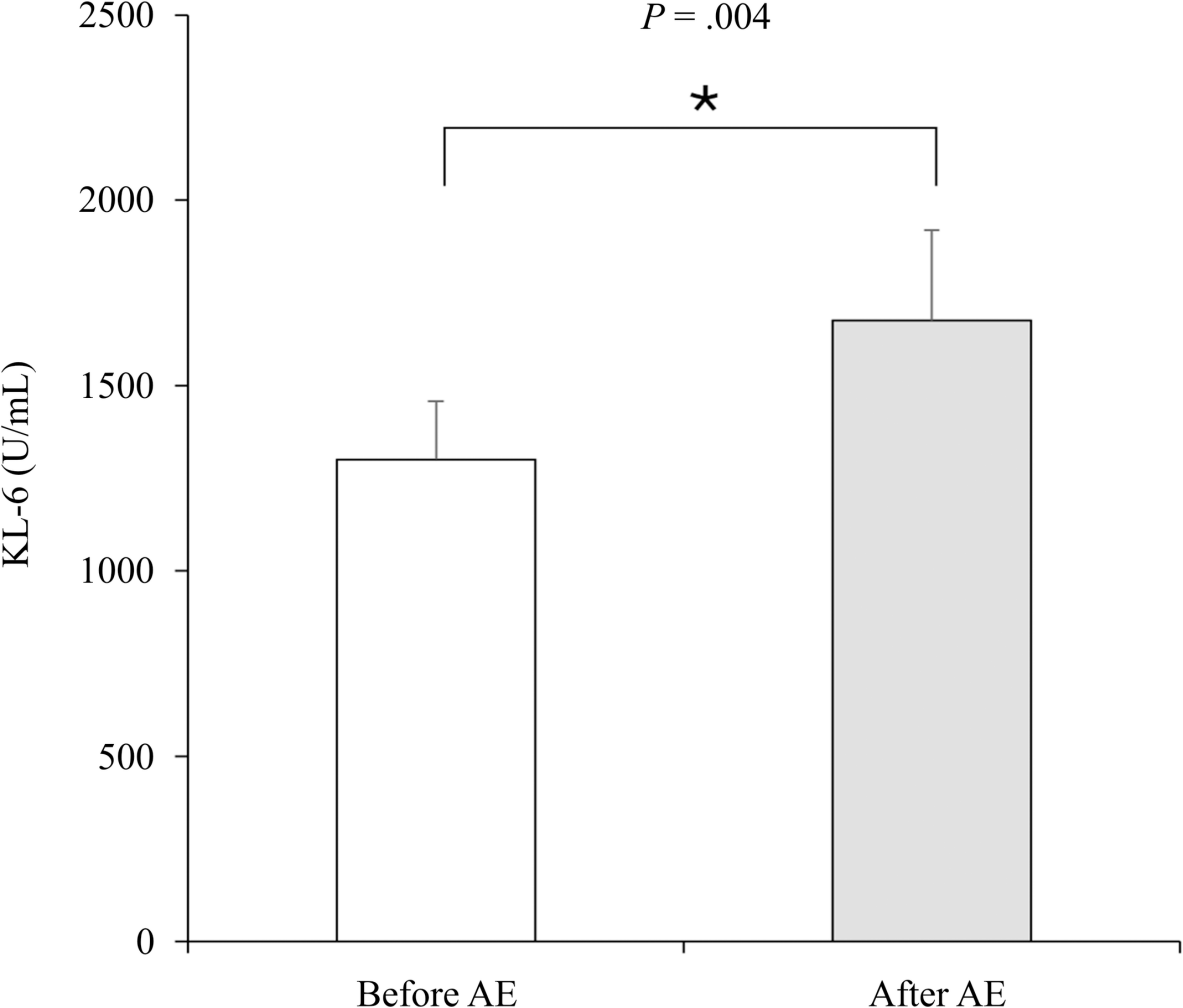
Comparative analysis of KL-6 values before and after AE in the AE group. AE, acute exacerbation; KL-6, Krebs von den Lungen-6.

When comparing KL-6 values between the pre-exacerbation phase and post-exacerbation phase in the AE group, the KL-6 values in the post-exacerbation phase were found to be significantly higher (Fig 3).

### Risk factors for AE-IPF

The difference between the KL-6 value immediately preceding the occurrence of AE and the baseline KL-6 value was proposed as the variable for logistic regression analysis aimed at identifying risk factors associated with AE occurrence. However, there were instances where the baseline KL-6 value was identical to the KL-6 value measured immediately prior to the occurrence of AE, making it impossible to conduct the analysis.

### Survival analysis

**Fig 4:**
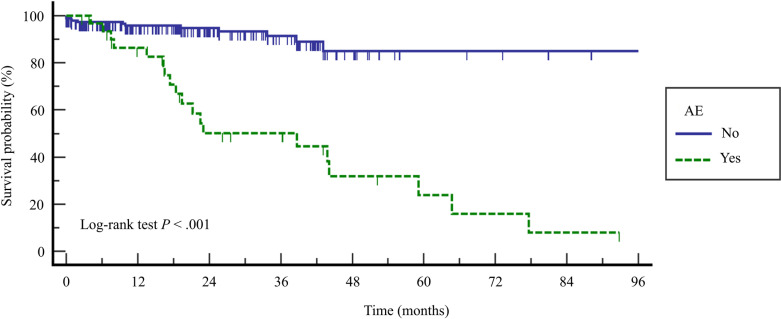
Comparison of the Kaplan-Meier survival curves according to occurrence of AE in patients with IPF. AE, acute exacerbation; IPF, idiopathic pulmonary fibrosis.

During the follow-up period, 31 patients (13.3%) died. In the AE group, the overall mortality rate was 57.6%. There was a significant difference in the 1-year (86.4% vs. 95.9%) and 3-year (50.2% vs. 91.4%) survival rates between the AE and non-AE groups (Fig 4). In survival analysis with the Cox proportional hazard regression model in all enrolled patients with IPF, the occurrence of AE (hazard ratio (HR) 74.09, 95% CI: 31.97–171.7, *P* < 0.001) and higher lactate dehydrogenase (LDH) (HR 1.02, 95% CI: 1.01–1.02, *P* < 0.001) were significant risk factors for mortality (Table 3).

**Table 3:** Survival analysis of prognostic factors for mortality in patients with IPF using Cox proportional hazards model.

	Univariate Analysis		Multivariate Analysis	
HR (95% CI)	*P*-value	HR (95% CI)	*P*-value
Age	1.01 (0.96-1.06)	.672		
Male	1.34 (0.47-3.84)	.587		
Ever smokers	0.79 (0.32-1.95)	.612		
BMI	0.95 (0.85-1.07)	.392		
Occurrence of AE	74.09 (31.97-171.70)	<.001	62.40 (20.20-192.72)	<.001
PFT, FVC, % predicted	0.96 (0.94-0.98)	.002	–	–
PFT, DLco, % predicted	0.97 (0.94-0.99)	.005	–	–
6MWD, m	1.00 (0.99-1.00)	.088		
6MWT, Baseline SpO_2_, %	0.85 (0.77-0.94)	.002	–	–
6MWT, Lowest SpO_2_, %	0.92 (0.88-0.97)	.001	–	–
WBC	1.00 (1.00-1.00)	.124		
LDH	1.01 (1.01-1.02)	<.001	1.02 (1.01-1.02)	<.001
CRP	1.07 (1.00-1.15)	.056		
BNP	1.00 (1.00-1.00)	.281		
KL-6 (U/mL)	1.00 (1.00-1.00)	.002	–	–
GAP stage Ⅰ	Reference			
GAP stage Ⅱ	1.96 (0.83-4.60)	.123		
GAP stage Ⅲ	9.09 (3.07-26.97)	.001	–	–
Home oxygen use	5.03 (2.36-10.69)	.001	–	–

IPF, idiopathic pulmonary fibrosis; HR, hazard ratio; CI, confidence interval; BMI, body mass index; AE, acute exacerbation; PFT, pulmonary function test; FVC, forced vital capacity; DLco, diffusing capacity of the lungs for carbon monoxide; 6MWD, six-minute walking distance; 6MWT, six-minute walk test; WBC, white blood cell; LDH, lactate dehydrogenase; CRP, C-reactive protein; BNP, brain natriuretic peptide; KL-6, Krebs von den lungen-6; GAP, gender, age, and physiology

## Discussion

In our study, KL-6 values were significantly higher in the AE group than in the non-AE group across all observed intervals, with notable differences observed in the patterns of change between the two groups. Furthermore, when comparing values before and after the acute exacerbation, KL-6 levels were significantly elevated in the post-exacerbation phase. The occurrence of AE and a higher baseline LDH value were independent risk factors for mortality in patients with IPF.

There has been a growing interest in the role of blood biomarkers as predictors of AE, supported by various evidences reported in the literature. Among the various known blood biomarkers, KL-6 has been widely used in clinical practice, with increasing evidence supporting its use [11,21]. Therefore, we focused on the role of KL-6 in predicting AE. The study by Oshimo et al. of 77 patients with IPF reported that baseline blood KL-6 level was an independent risk factor of AE (HR: 1.001, 95% CI: 1.000–1.001, p = 0.010), and the best cut-off level was 1300 U/mL [24]. Huang et al. performed a study of 57 IPF patients on nintedanib and reported that higher baseline KL-6 (HR 4.52, 95% CI: 1.63–12.55, p = 0.004) was a significant risk factor for AE [33]. The finding in our study that baseline KL-6 levels were significantly higher in the AE group compared to the non-AE group aligns with results from previous studies. Furthermore, our research demonstrated that the trends of KL-6 changes over time in the AE group exhibited significant differences compared to the patterns observed in the non-AE group. This suggests that changes in KL-6, particularly upward trends, may be associated with an increased risk of AE. The clinical significance of KL-6 changes is further corroborated by the study conducted by Choi et al., which reported that changes in KL-6 levels could serve as significant factors in the clinical course of patients with AE-IPF [34]. Our findings suggest that not only the baseline KL-6 value but also the continuous changes in KL-6 values during the follow-up period can be utilized as a tool for predicting. Our findings suggest that both the baseline KL-6 value and the trend of changes in KL-6 values during the follow-up period can be utilized as tools for predicting AE; however, further research is needed to determine and validate a cut-off value that indicates significant changes for predicting AE-IPF.

The comparison of KL-6 values before and after AE revealed that post-exacerbation values of KL-6 were significantly higher. This finding suggests that elevated KL-6 levels following AE may serve as risk factors for AE-IPF recurrence, disease progression, and mortality. In our study, among the 33 patients in the AE group, 16 (48.5%) patients experienced recurrence of AE-IPF. Additionally, among the 19 patients who died, 10 (50.3%) patients had mortality related to recurrent AE-IPF, indicating that the increase in KL-6 values after AE is associated with a trend towards poor prognosis. This aligns with the findings of Choi et al., which demonstrated that patients hospitalized with AE-IPF who had KL-6 values that increased by more than 10% at one-week intervals exhibited significantly higher in-hospital mortality rates (63.2% vs. 6.1%) compared to those without such increases [34]. In our study, LDH was a risk factor for the mortality of IPF patients along with the occurrence of AE-IPF. A previous study by Kishaba et al. showed similar results [35]. In 65 patients with IPF, both LDH at diagnosis of AE (HR 1.003, 95% CI: 1.001–1.005, p = 0.004) and change in LDH (HR 1.004, 95% CI: 1.001–1.008, p = 0.017) were independent risk factors for mortality in multivariable analysis, while change in KL-6 (HR 1.000, 95% CI: 0.999–1.001, p = 0.197) was not. Additional research is needed to further assess the role of LDH as prognostic factor for mortality in AE-IPF.

This study has several limitations. First, it was a retrospective study with a small number of only Korean patients, which may limit the generalizability of our findings. However, the baseline characteristics and results were similar to those of patients in other studies [5,6,36,37]. Second, in this study, baseline was defined as a stable state without AE rather than a specific time point such as IPF diagnosis or initiation of treatment. This inconsistency might have resulted in selection bias. Further prospective research is needed. Third, certain instances were identified during analysis where calculating the difference between the baseline KL-6 values and the KL-6 values immediately preceding AE was not feasible. This limitation hindered our ability to analyze the clinical significance of the absolute change in KL-6, which constituted the fundamental objective of the study. Nonetheless, we observed that baseline KL-6 values were significantly elevated in the AE group compared to the non-AE group, revealing a consistent trend of significant differences maintained across all intervals. This finding aligns with existing literature that suggests higher baseline KL-6 values correlate with an increased risk of AE-IPF. Furthermore, the temporal changes in KL-6 values exhibited significant differences between the AE and non-AE groups, indicating that these changes may possess clinical relevance as a predictor of AE. However, further research is needed to elucidate the role of quantitative changes in KL-6 and to identify and validate the optimal cut-off value for prediction of AE-IPF.

## Conclusions

In conclusion, the findings of our study suggest that patients exhibiting elevated baseline KL-6 values are at an increased risk for AE-IPF. Moreover, the observed patterns of change in KL-6 values during the follow-up period highlight their potential utility as a predictive tool for AE-IPF. Consequently, we suggest the implementation of serial measurements of KL-6 values in clinical practice to enhance the prediction of AE-IPF.
